# Sex differences in sleep and memory relationships in older adults at risk for Alzheimer’s disease

**DOI:** 10.3389/fendo.2026.1930177

**Published:** 2026-10-12

**Authors:** Negin Sattari, Abhishek Dave, Ivy Y. Chen, Kitty K. Lui, Miranda G. Chappel-Farley, Destiny E. Berisha, Kate E. Sprecher, Brady A. Riedner, Stephanie Jones, Barbara B. Bendlin, Bryce A. Mander, Ruth M. Benca

**Affiliations:** 1 Department of Psychiatry and Human Behavior, University of California, Irvine, Irvine, CA, United States; 2 Department of Cognitive Sciences, University of California, Irvine, Irvine, CA, United States; 3 San Diego State University/University of California San Diego Joint Doctoral Program in Clinical Psychology, San Diego, CA, United States; 4 Department of Psychiatry, University of Pittsburgh School of Medicine, Pittsburgh, PA, United States; 5 Department of Neurobiology and Behavior, University of California, Irvine, Irvine, CA, United States; 6 Department of Psychiatry, Wisconsin Institute for Sleep and Consciousness, University of Wisconsin-Madison, Madison, WI, United States; 7 Department of Medicine, Division of Geriatrics and Gerontology, University of Wisconsin-Madison, Madison, WI, United States; 8 Department of Psychiatry and Behavioral Medicine, Wake Forest University School of Medicine, Winston-Salem, NC, United States

**Keywords:** Alzheimer’s disease, APOE ϵ4, cognitive aging, NREM sleep, sex differences, sleep spindles, sleep-dependent memory consolidation, slow wave activity

## Abstract

**Introduction:**

Biological sex is an important modifier of aging and Alzheimer’s disease risk, yet the sleep-related mechanisms through which sex and genetic risk interact to influence memory remain poorly understood. We examined whether associations among non-rapid eye movement sleep physiology, overnight memory retention, and apolipoprotein E ϵ4 (*APOE* ϵ4) status, a genetic risk factor for Alzheimer’s disease, differed by sex in cognitively unimpaired older adults enriched for Alzheimer’s disease risk.

**Methods:**

Eighty participants completed overnight polysomnography with high-density electroencephalography, and 65 also completed an evening encoding/immediate recall and morning delayed recall word-pair memory task.

**Results:**

Overnight memory retention did not differ by sex. However, exploratory analyses of the proportional overnight retention measure suggested a sex × *APOE* ϵ4 interaction, such that female APOE ϵ4 carriers may exhibit reduced overnight forgetting relative to female non-carriers and male carriers. Although counterintuitive, this pattern may reflect compensatory or resilience-related sleep-memory mechanisms in cognitively unimpaired women at genetic risk for Alzheimer’s disease rather than a protective effect of *APOE* ϵ4 itself. Women spent more time in N3 sleep and had higher non-rapid eye movement delta and slow sigma power than men. Sleep-memory associations also differed by sex: greater N2 duration was more strongly associated with better overnight retention in men, whereas greater frontal slow sigma power and fronto-central fast sigma power were positively associated with retention in women. Exploratory APOE-stratified analyses suggested sex-specific differences in sleep stage-memory relationships, with more positive N2-memory associations in male than female APOE ϵ4 carriers and more positive N3-memory associations in female than male carriers; both differences remained significant after FDR correction. TFCE-corrected analyses further identified positive associations between NREM delta and sigma activity and overnight memory retention in female APOE ϵ4 carriers.

**Discussion:**

These findings highlight sex-specific patterns in NREM sleep physiology and sleep-memory relationships in cognitively unimpaired older adults at risk for Alzheimer’s disease. More broadly, the results suggest that sex and *APOE* ϵ4 status may jointly shape the neurophysiological pathways linking sleep and memory, underscoring the importance of considering sex as a biological variable when studying sleep, cognitive aging, and Alzheimer’s disease vulnerability.

## 1 Introduction

Alzheimer’s disease (AD) is the most common cause of dementia and a major global health concern, with cases increasing as the population ages (1). Importantly, biological sex is a major, yet often underexamined, factor influencing cognitive aging and AD risk (2–4). Additionally, the apolipoprotein E (*APOE*) *ϵ4* allele is a well-established and non-modifiable genetic risk factor for late-onset sporadic AD, associated with earlier AD onset, greater amyloid (5) and tau burden (6), and accelerated neurodegeneration and cognitive decline relative to *ϵ4* non-carriers (3, 7, 8). Although *APOE ϵ4* status and biological sex are both important contributors to AD risk, the biological pathway through which they interact to influence vulnerability, particularly in asymptomatic individuals, remains incompletely understood (2, 9). Epidemiological studies suggest that female *ϵ4*-carriers show faster cognitive decline, steeper trajectories of brain atrophy, and greater tau accumulation (10) compared to male carriers (11, 12). Together, these findings suggest that sex may meaningfully modify the effects of *APOE ϵ4* on aging-related outcomes.

Sleep is one candidate pathway through which sex and *APOE ϵ4* may influence cognitive aging and AD-related vulnerability. Several studies indicate that sleep influences cognitive health and may contribute to AD pathogenesis (13–15), and some evidence indicates that there may be sex differences in how sleep changes as AD progresses (16). Early work in AD populations demonstrated reduced slow wave sleep (SWS), reduced REM sleep, and increased nighttime wakefulness, suggesting that sleep architecture is altered in AD (16). These changes differed between men and women, with women showing higher SWS duration than men regardless of AD stage, although the study (16) did not formally assess whether AD-related sleep alterations interacted with sex beyond normative aging effects.

Non-rapid eye movement (NREM) sleep plays an important role in the consolidation of newly acquired information into long-term memory (17, 18). Specific NREM oscillations, particularly slow waves and sleep spindles, are thought to support this process through hippocampal-cortical communication (17, 19, 20). Importantly, these oscillatory features also exhibit robust age- and sex-related differences across adulthood, including differences in slow wave and spindle expression between men and women (21–23). Decreases in slow wave and sleep spindle amplitude appear particularly prominent in older men, and disruptions in these oscillations have been reported early in the course of AD and linked to impaired memory consolidation (24, 25).

Beyond their role in memory consolidation, NREM sleep oscillations have also been implicated in AD pathophysiology and may represent one mechanism linking sleep disruption with cognitive vulnerability in aging (25, 26). *APOE ϵ4*, the strongest genetic risk factor for sporadic, late-onset AD, has similarly been associated with alterations in sleep and memory processes (16, 27). Together, these findings suggest that sleep may represent one mechanism through which biological sex and genetic risk contribute to variability in cognitive aging and AD vulnerability (25, 28).

Biological sex is an important source of variability in sleep neurophysiology and memory, and genetic risk factors may further modify these relationships. Presence of the *APOE ϵ4* allele has been associated with reduced slow wave sleep and altered memory performance in cognitively unimpaired older adults (16, 27), suggesting that genetic risk may contribute to variability in sleep-memory relationships. Although *APOE ϵ4*-related alterations in sleep architecture have been reported in large population-based cohorts, including increased N3 sleep duration among *ϵ4* homozygous men (29), less is known about how *APOE ϵ4* interacts with biological sex to affect sleep physiology and sleep-memory relationships in cognitively unimpaired older adults (14). Much of the existing literature examining effects of *APOE ϵ4* status on sleep neurophysiology either collapsed across sex, treated sex only as a covariate, or relied on predominantly male samples, limiting insight into potential sex-specific sleep-genetic interactions. This limitation is particularly important given robust sex differences in sleep physiology and evidence that women may exhibit greater *APOE ϵ4*-related AD vulnerability (14, 30).

Women, in addition to bearing a disproportionate lifetime risk for AD, also exhibit higher prevalence of sleep disturbances in midlife and older adulthood, including insomnia symptoms and sleep fragmentation (31). Prior work demonstrates robust sex differences in NREM electroencephalography (EEG) measures across adulthood. In general, women show greater slow wave activity and higher sigma/spindle-related activity than men, whereas men show more pronounced age-related reductions in slow wave and spindle expression (21, 32, 33). Given the established role of NREM oscillations in memory consolidation (15, 16), these sex-related differences in sleep physiology may have functional implications for sleep-dependent retention of episodic memory in older adults. Consistent with this possibility, prior studies in younger cohorts demonstrated that sleep-dependent memory consolidation differs by sex and hormonal status. For example, nap-related improvements in declarative and motor memory have been reported to be more pronounced during the luteal relative to follicular phase in women, and these improvements were associated with increased spindle activity (32). In perceptual learning, women showed more generalized post-sleep improvement, whereas men showed improvement that was more specific to the trained stimulus (34). Together, these findings provide a rationale for examining whether relationships among NREM sleep, overnight sleep-dependent memory retention, and *APOE ϵ4* status differ by sex in cognitively unimpaired older adults.

We addressed these knowledge gaps in the present study by implementing polysomnography (PSG) with high-density EEG and an overnight word-pairs memory task to test whether associations between NREM sleep architecture (i.e., N2 and N3 sleep stages), NREM sleep EEG spectral activity (e.g., slow wave activity and sigma power), and overnight memory retention differ across sex and *APOE ϵ4* status groups. We hypothesized that sex would be associated with differences in sleep architecture and would show different relationships with overnight memory retention. We further explored whether *APOE ϵ4* status modifies sex-related effects.

## 2 Materials and methods

### 2.1 Participants

Eighty cognitively unimpaired older adults completed an overnight polysomnography (PSG) study with high-density electroencephalography (hdEEG; mean age=61.3 ± 5.1 years; 49 female; 25 *APOE ϵ4* carriers,14 female). This cohort was utilized to examine associations between sex, APOE genotype, and sleep. A subset of these participants (N = 65; mean age=61.12 ± 5.9 years; 40 female; 20 *APOE ϵ4* carriers) additionally completed the word-pairs task to assess overnight memory retention. This subset was utilized to examine effects of sex, APOE genotype, and sleep expression on overnight sleep-dependent memory retention. Participants were recruited from the Wisconsin Alzheimer’s Disease Research Center (ADRC) clinical core. The cohort was enriched for AD risk (35, 36) characterized by parental history and *APOE ϵ4* positivity (**Table 1**). The APOE ϵ4 carrier group was predominantly composed of ϵ3/ϵ4 heterozygotes; only two participants were ϵ4/ϵ4 homozygotes. The small number of homozygotes precluded genotype dose-response analyses. Individuals with significant neurological, psychiatric, or medical conditions that could affect cognition or sleep were excluded. Recruitment was not stratified or targeted based on APOE genotype, and APOE status was determined following enrollment and was not used as a selection criterion for participation. Additionally, use of medications known to influence sleep or EEG (e.g., antipsychotics, sedatives, SSRIs) was exclusionary. Participants were classified as cognitively unimpaired through the Wisconsin ADRC clinical evaluation, which included comprehensive neuropsychological assessment using the National Alzheimer’s Coordinating Center Uniform Data Set (UDS) (37) and additional validated assessments (38, 39). Neuropsychological performance of the present sample is summarized in **Supplementary Table S2**. All participants provided written informed consent, and study procedures were approved by the Institutional Review Board at the University of Wisconsin-Madison.

**Table 1:** Participant characteristics.

Characteristic	Sleep physiology sample (N = 80)	Female, N = 49 (Male, N = 31)	Sleep-memory sample (N = 65)	Female, N = 40 (Male, N = 25)
Demographics				
Age, years	61.3 ± 5.1	60.69 ± 5.19 (62.99 ± 7.00)	61.1 ± 5.0	60.24 ± 4.85 (62.53 ± 7.17)
Female, n (%)	49 (61.3%)		40 (61.5%)	
Education, years	16.40 ± 2.40	15.77 ± 0.32 (17.38 ± 2.33)	16.6 ± 2.28	16.1 ± 2.15 (17.56 ± 2.23)
Body mass index (kg/m^2^)	28.10 ± 5.60	27.60 ± 6.05 (28.90 ± 4.79)	27.73 ± 5.18	26.61 ± 5.07 (28.91 ± 4.96)
Genotype				
APOE ϵ4 carriers, n (%)	25 (31.3%)	14 (11)	20 (30.8%)	10 (10)
Female ϵ4 carriers, n (%)	14 (17.5%)		12 (18.5%)	
APOE ϵ4 homozygous carriers, n (%)	2 (2.5%)	1 (1)	2 (3.07%)	1 (1)
	2 (2.5%)	1 (1)	2 (3.07%)	1 (1)
Sleep-related measures				
Apnea-hypopnea index (events/h)	8.43 ± 12.44	4.5 ± 0.97 (14.46 ± 16.41)	7.9 ± 10.9	4.19 ± 4.84 (13.74 ± 14.77)
N1 sleep, % TST	0.09 ± 0.09	0.07 ± 0.05 (0.31 ± 0.13)	0.08 ± 0.07	0.06 ± 0.04 (0.11 ± 0.09)
N2 sleep, % TST	0.60 ± 0.10	0.25 ± 0.01 (0.62 ± 0.10)	0.61 ± 0.11	0.59 ± 0.11 (0.63 ± 0.09)
N3 sleep, % TST	0.13 ± 0.10	0.16 ± 0.11 (0.08 ± 0.07)	0.13 ± 0.10	0.15 ± 0.11 (0.09 ± 0.07)
REM sleep, % TST	0.17 ± 0.06	(0.17 ± 0.06 (0.15 ± 0.06)	0.18 ± 0.06	0.18 ± 0.06 (0.16 ± 0.05)

The sleep physiology sample (N = 80) includes all participants with valid overnight EEG data. The sleep-memory sample (N = 65) includes participants with complete overnight memory data. Demographic and clinical characteristics were comparable across samples. All reported means are presented with their corresponding standard deviations (mean ± SD).

### 2.2 Study protocol

Participants completed an in-laboratory evening session of memory encoding followed by an immediate recall test, overnight polysomnography with hdEEG, and a delayed recall test the following morning to assess overnight memory retention. APOE genotype was determined from whole blood samples using DNA extraction followed by competitive allele-specific PCR-based KASP genotyping for rs429358 and rs7412, as previously described (40). The final analytic sample consisted of 80 participants with PSG data and 65 participants with memory data at both immediate and delayed recall testing sessions.

### 2.3 Polysomnography and EEG acquisition

Overnight sleep was assessed using standard clinical polysomnography (PSG) and concurrently recorded hdEEG (41). PSG data were collected using a customized Alice 5 System (Philips Respironics, Murrysville, PA, USA) and included electrooculogram (EOG), submental electromyogram (EMG), electrocardiogram (ECG), respiratory inductance plethysmography, pulse oximetry, and body position monitoring. Simultaneously, 256-channel hdEEG was recorded using a NetAmps 300 amplifier and NetStation software (Electrical Geodesics Inc., EGI), with vertex referencing. EEG signals were digitized at 500 Hz. Sleep staging was performed by a registered polysomnographic technologist according to American Academy of Sleep Medicine (AASM) criteria (34) using six standard 10–20 EEG derivations (F3, F4, C3, C4, O1, O2) extracted from the hdEEG montage and re-referenced to the contralateral mastoid. Sleep scoring was conducted using Alice Sleepware and reviewed in full by a board-certified sleep physician (co-author R.M.B.), including verification of clinical sleep events. Standard sleep architecture measures were derived, including total recording time (TRT), total sleep time (TST), sleep latency (SL), sleep efficiency (SE), wake after sleep onset (WASO), and percentage of time spent in each sleep stage (N1, N2, N3, REM). In addition, total sleep apnea-hypopnea index (AHI; number of apneas and hypopneas per hour), which is a clinical measure characterizing sleep-disordered breathing disorders, was also derived (42, 43).

### 2.4 EEG spectral analysis

Preprocessing and spectral analyses were conducted using custom MATLAB scripts with EEGLAB toolboxes (http://sccn.ucsd.edu/eeglab). As previously described (41), EEG data were notch-filtered in the 59.5-60.5 Hz range to remove line noise and then bandpass-filtered between 0.3–55 Hz using a 5500-point Hamming-windowed sinc Finite Impulse Response filter for both operations. The 50 outermost electrodes were excluded from further analyses due to recurring physiological artifacts at these locations across participants. The remaining 206 electrodes were average-referenced and manually inspected for persistent, long duration noise to remove compromised channels. Data corresponding to NREM sleep stages (N2 and N3) were then extracted from these electrodes and concatenated into separate matrices for subsequent analyses. A three-stage semi-automated artifact rejection procedure was subsequently applied to eliminate short durations of widespread noise and consistently noisy EEG derivations (44–46). Epochs with arousals were first removed. Next, an automated 99.8th-percentile threshold on broadband power was applied to exclude high-amplitude artifacts. Residual short duration artifacts and noisy electrodes were then visually rejected. Finally, deleted channels were reconstructed using spherical spline interpolation, yielding clean NREM EEG data for all 80 subjects (47, 48). Next, spectral analyses were performed using a multitaper Fast Fourier Transform on cleaned EEG data to estimate absolute and relative power from 0.5–40 Hz. Eleven Slepian tapers (bandwidth = 0.25 Hz) were applied with zero padding and 30s windows sliding every 5s. The resulting time-frequency matrix (μV^2^/Hz) was integrated and normalized to total power within each channel to derive relative spectral power (unitless ratio), indicating the proportion of power within each frequency band. Median spectral power was computed across NREM epochs for each of the 1,294 frequency bins, then averaged within canonical frequency bands: slow oscillation: 0.5-1, delta:1-4.5, theta: 4.5-7.5Hz, alpha: 7.5-11Hz, slow sigma: 11-13Hz, fast sigma: 13-16Hz, beta: 16-28Hz, and gamma: 28-40Hz). Spectral analyses were performed using the Chronux v2.12 toolbox (http://chronux.org) in MATLAB. Analyses focused *a priori* on slow wave activity (i.e., slow oscillation and delta) and sigma frequency bands because of the relevance of slow waves and sleep spindles for memory consolidation and the known impacts of sex, aging, and AD on these oscillations (17–19).

### 2.5 Word-pairs memory task

Overnight episodic memory retention was assessed using a validated word-pairs task that is sensitive to sleep-dependent memory consolidation in both young adults and individuals with mild cognitive impairment (MCI) (49–52). The task was administered using E-Prime software (Psychology Software Tools, Pittsburgh, PA) and consisted of three phases: encoding, immediate recall, and delayed recall. The encoding phase occurred in the evening prior to sleep onset. During this phase, participants were presented with 88 semantically unrelated word-pairs, each displayed sequentially for four seconds with one word positioned above the other at the center of a computer monitor. Participants were instructed to learn the word-pairs for later recall. To minimize primacy and recency effects, the first and last four word-pairs were excluded from subsequent recall phases. Immediately following encoding, participants completed an immediate recall phase in which one word from each pair was presented individually on the screen, and participants were instructed to type the corresponding word. Responses were self-paced, and word-pair order was randomized across participants and task phases. Participants were seated with the experimenter out of view to minimize feedback-related bias. After completion of the immediate recall phase, participants were shown feedback indicating their percentage accuracy. Encoding and immediate recall phases were repeated until participants achieved a minimum of 60% accuracy; performance from the final immediate recall phase was used for analysis. The delayed recall phase was administered the following morning, approximately one hour after sleep offset, and was completed only once without performance feedback. Accuracy during both immediate and delayed recall phases was calculated as the proportion of correctly recalled word-pairs. Responses containing clear typographical errors (e.g., forg instead of frog) were corrected offline prior to analysis. Overnight memory retention was quantified as the proportional change in recall performance from immediate to delayed testing, calculated as (Delayed Recall-Immediate Recall)/Immediate Recall. To address non-normality in the distribution of this variable, a cube-root transformation was applied to this proportional change prior to statistical analyses.

### 2.6 Data reduction

Participants were included in sleep-dependent memory analyses if both immediate and delayed recall data were available. Fifteen participants were excluded from memory analyses due to missing memory data (n = 11) or identification as statistical outliers (n = 4). Outliers were defined *a priori* as values exceeding ±3 standard deviations from the mean of the overnight memory retention metric. Participants failing to meet the ≥60% performance criterion at immediate recall were excluded prior to these analyses. To assess potential selection bias in the memory analytic sample, participants included in memory analyses were compared with those excluded from memory analyses on age, sex, and APOE ϵ4 status. Age was compared using an independent-samples t-test, whereas sex and APOE ϵ4 status were compared using Fisher’s exact tests. No significant differences were observed (all p > 0.21).

### 2.7 Statistical analysis

Analyses followed a hierarchical approach. We first examined main effects of sex on memory and sleep outcomes. We then conducted exploratory models including *APOE ϵ4* status and sex×*APOE ϵ4* interactions to examine whether genetic risk may further modify observed sex-related patterns. A univariate ANCOVA was first conducted to examine overnight memory retention, with overnight memory retention as the dependent variable and sex as the fixed factor. Age, apnea-hypopnea index (AHI), and years of education included as covariates given their established associations with sleep physiology and cognitive performance in older adults (10). Follow-up exploratory models added *APOE ϵ4* status and the sex×APOE *ϵ4* interaction. These analyses included participants with available immediate and delayed recall data (n=65). To evaluate whether the proportional overnight retention measure was influenced by immediate-recall performance, we conducted additional sensitivity analyses. First, we examined the association between immediate recall and proportional overnight retention. Second, the primary ANCOVA was repeated with immediate recall included as an additional covariate. Third, immediate recall was examined as the dependent variable in an ANCOVA including sex, APOE ε4 status, their interaction, age, AHI, and years of education. Full results of these sensitivity analyses are reported in the Supplementary Results. Similar ANCOVA models were conducted to examine associations by sex and exploratory analyses by *APOE ϵ4* status with sleep continuity measures, including wake after sleep onset (WASO) and sleep efficiency (SE). Further, a multivariate ANCOVA was first conducted to examine effects of sex on sleep architecture, with percentage of time spent in each sleep stage as dependent variables, sex as the fixed factors, and age, education, and AHI as covariates. Follow-up exploratory models added *APOE ϵ4* status and the sex×APOE *ϵ4* interaction. These analyses included all participants with PSG data (n = 80). Age, years of education, and AHI were included as covariates given their known associations with sleep architecture and cognitive performance and because they did not differ systematically across sex or *APOE ϵ4* groups. Significant interactions were followed by planned *post hoc* comparisons using least significant difference tests to examine simple effects within sex and *APOE ϵ4* groups. *Post hoc* analyses were conducted only when omnibus interaction terms reached statistical significance. Given the modest sample size, particularly within sex-by-APOE ϵ4 subgroups, sensitivity power analyses were conducted to estimate the minimum interaction effects detectable with the available samples. Assuming a two-sided α of.05 and 80% power, the full sleep sample of 80 participants provided sufficient power to detect an interaction effect of approximately Cohen’s f = .32 (partial η^2^ ≈.09), whereas the memory-analysis sample of 65 participants provided sufficient power to detect a sex × APOE ϵ4 interaction of approximately Cohen’s f = .35 (partial η^2^ ≈.11). Thus, the study was primarily powered to detect medium-to-large interaction effects and may have been underpowered to detect smaller effects. To account for multiple testing across sleep stages, Benjamini–Hochberg false discovery rate (FDR) correction was applied across the four sleep stages (N1, N2, N3, and REM) within each family of related correlation analyses. FDR correction was similarly applied across the corresponding Fisher r-to-z tests comparing correlation coefficients between groups. The same procedure was applied to the exploratory APOE ϵ4-stratified analyses. Raw p-values and FDR-adjusted q-values are reported for findings reaching nominal significance. For sleep EEG spectral power analyses assessing associations with overnight changes in word-pair memory performance across groups, Pearson’s r values were first computed at each electrode across the scalp. Electrode-wise analyses were corrected for multiple comparisons using threshold-free cluster enhancement (TFCE) with 5,000 permutations, using E = 0.5 and H = 2.0, with statistical significance set at p <.05. All TFCE tests were two-sided (53–55). Absolute spectral power from electrodes that remained significant after TFCE correction was extracted and averaged within each significant cluster. Partial correlations were then used to examine associations between averaged spectral power and overnight memory retention while adjusting for age, years of education, and AHI. In sensitivity analyses, these partial-correlation analyses were repeated with TST included as an additional covariate. To evaluate group differences in spectral power, independent-samples t-tests were conducted at each electrode, followed by TFCE correction (5,000 permutations) for multiple comparisons across topography. TFCE correction for multiple comparisons analysis was implemented using a custom MATLAB (R2017b; MathWorks, Natick, MA, USA) derived from previously described nonparametric permutation methods (53–55). EEG preprocessing and data handling relied on EEGLAB (56).

All analyses were conducted using SPSS (Version 28.0.0.0) (IBM SPSS Statistics, Inc., Chicago, IL).

## 3 Results

### 3.1 Sample characteristics, data completeness, and potential selection bias

Of the 80 participants who completed overnight polysomnography, 65 completed the memory task. To assess potential selection bias, participants included in memory analyses were compared to those excluded, based on age, sex, and *APOE ϵ4* status. No significant differences were observed between included and excluded participants (all p values >.21), indicating that the analytic sample was not systematically biased by which individuals completed memory testing.

### 3.2 Sex, *APOE ϵ4* status, and overnight memory retention

We first examined whether overnight sleep-dependent memory retention differed by sex. We found no significant main effect of sex on memory retention, F(1,58) = .13, p = .72, partial η^2^ = .002. No significant effects of age (p = .37), AHI (p = .48), or years of education (p = .11) were observed, **Supplementary Figure 1**. The overall model was not significant, F(4,58) = 1.07, p = .38, R^2^ = .07. We next conducted exploratory analyses examining whether *APOE ϵ4* status may modify the relationship between sex and overnight memory retention. A follow-up ANCOVA examining the proportional overnight retention score, including sex, *APOE ϵ4* status, and their interaction revealed a significant sex×*APOE ϵ4* interaction on overnight memory retention, F(1, 58) =7.36, p =.009, partial η^2^ =.116. No significant main effects of sex (p = .26) or APOE *ϵ4* status (p = .88) were observed, **Figure 1**. *Post hoc* comparisons confirmed no difference in overnight memory retention between *APOE ϵ4* carriers and non-carriers among men [t(23) =.91, p=.37], **Figure 1**. Among women, the proportional retention analysis indicated less overnight forgetting in APOE ϵ4 carriers compared with non-carriers [t(38) =2.52, p=.04]. Furthermore, male *APOE ϵ4* carriers demonstrated poorer memory retention compared to female carriers [t(18) =-2.11, p=.05], **Figure 1**. These exploratory findings suggest that proportional overnight retention may vary as a function of sex and APOE ϵ4 status, but should be interpreted cautiously given the modest subgroup sizes and sensitivity of the effect to how memory change is operationalized. The sex × APOE ϵ4 interaction remained significant in a sensitivity analysis excluding the two ϵ4/ϵ4 homozygous participants, F(1,56) = 4.64, p = .036, partial η^2^ = .078 (**Supplementary Table S3**). Additional sensitivity analyses indicated that the sex × APOE ε4 interaction was not explained by systematic differences in immediate-recall performance. Immediate recall was not significantly associated with proportional overnight retention (r = .11, p = .39). Moreover, when immediate recall was included as an additional covariate in the primary ANCOVA, the sex × APOE ε4 interaction remained significant, F(1,56) = 4.05, p = .049, partial η² = .067, whereas immediate recall was not significant, F(1,56) = 0.10, p = .755, partial η² = .002. Immediate recall itself also did not significantly differ by sex, APOE ε4 status, or their interaction (Supplementary Results).

**Figure 1:**
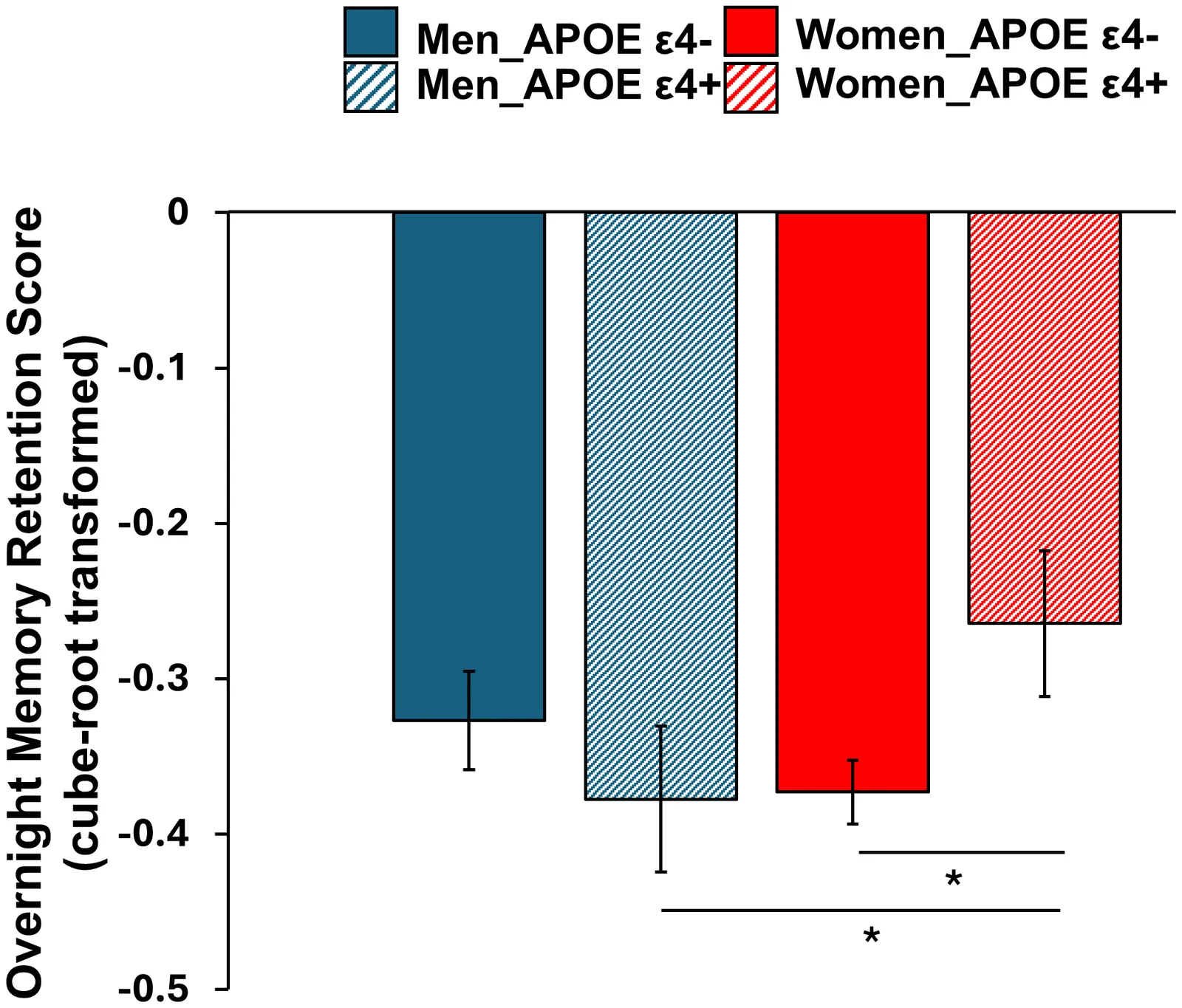
Overnight sleep-dependent memory retention as a function of sex and *APOE ϵ4* status. Solid bars represent *APOE ϵ4* non-carriers and hatched bars represent *APOE ϵ4* carriers. Blue bars denote men and red bars denote women. Bars show group means, and error bars represent ± SEM. Overnight memory retention was calculated as proportional change from immediate to delayed recall [(Delayed Recall − Immediate Recall)/Immediate Recall] and cube-root transformed to address non-normality. Values displayed in the figure represent the cube-root-transformed proportional overnight retention scores, which were also used in the inferential statistical analyses. Asterisks (*) indicate significant group differences (p <.05).

### 3.3 Sex, *APOE ϵ4* status, and sleep architecture

The multivariate ANCOVA, conducted on percentage of time spent in N1, N2, N3, and REM sleep stages revealed significant overall model effects for N1 (p<.001), N3 (p=.002), and REM sleep (p=.03). There was a significant main effect of sex on N3, F(1,75) = 7.81, p = .007, partial η^2^=.09, indicating that women spent more time in N3, consistent with prior literature (57). Further, we observed a main effect of age on N1 (p<.001) indicating older age was associated with spending more time in N1 sleep, **Supplementary Figure 2**. There were no other main effects of age on any other sleep stages (all ps>.055), and no significant effects of AHI were detected (all p values>.07). We next explored whether *APOE ϵ4* status moderated sex differences in sleep architecture. A similar multivariate ANCOVA model revealed a significant overall model effect for N1 (p <.001), N2 (p = .03), and N3 sleep (p = .006), but not for REM sleep (p = .125), **Figure 2**. For N1 sleep, older age was associated with increased N1 sleep percentage, F(1,73) = 15.36, p <.001, partial η^2^ = .17, and main effects of age were not observed for any other sleep stage (all ps >.06). We observed a main effect of sex for N2, F(1,73) = 5.16, p = .03, partial η^2^ = .07 indicating men spent more time in N2, and for N3, F(1,73) = 9.41, p = .003, partial η^2^ = .11 indicating women spent more time in N3 sleep. No significant main effects of *APOE ϵ4* status on sleep stage duration were found (all ps>.64). In addition, the sex×APOE ϵ4 interaction showed a trend-level effect but did not reach statistical significance, F(1,73) = 3.87, p = .053. Descriptive exploratory comparisons indicated that among APOE ϵ4 non-carriers, women, compared with men, spent less time in N1 (p <.001) and more time in N3 (p = .02); however, these subgroup comparisons should be interpreted cautiously given the nonsignificant interaction. Further, among *APOE ϵ4* carriers, women, compared to men, spent less time in N2 (p=.007), and more time in N3 (p=.007), **Figure 2**. Together, these findings suggest that sex differences in sleep architecture are robust, particularly for deeper NREM sleep stages, with women consistently exhibiting greater N3 sleep duration and men showing relatively greater N2 sleep duration. Although descriptive subgroup patterns differed across APOE ϵ4 groups, the sex × APOE ϵ4 interaction did not reach statistical significance. Therefore, the present findings do not provide conclusive evidence that APOE ϵ4 status modifies sex differences in sleep architecture. We next examined whether sex was associated with measures of sleep continuity, including wake after sleep onset (WASO) and sleep efficiency (SE). We found a significant main effect of age F(1,75) = 8.65, p = .004, partial η^2^ = .103, such that older age was associated with increased WASO. No significant effects of sex (p = .38) or AHI (p = .51) were detected. When analysis was further stratified by *APOE ϵ4* status, a significant main effect of age persisted, F(1,73) = 9.04, p = .004, partial η^2^ = .110. However, no significant main effects of sex (p = .47) or *APOE ϵ4* status (p = .43) were observed. In addition, no significant sex×*APOE ϵ4* interaction was found, F(1,73) = 0.01, p = .90, partial η^2^ <.001. For SE, a significant main effect of age was observed, F(1,75) = 6.98, p = .010, partial η^2^ = .085, indicating lower sleep efficiency with increasing age. No significant effects of sex (p = .75) or AHI (p = .98) were detected. Next, when the analysis was further stratified by *APOE ϵ4* status, a significant main effect of age persisted, F(1,73) = 6.96, p = .01, partial η^2^ = .08. No significant main effects of sex (p = .91) or *APOE ϵ4* status (p = .75) were detected. In addition, no significant sex×*APOE ϵ4* interaction was observed, F(1,73) = 0.16, p = .69, partial η^2^ = .002. Together, these findings indicate that sex differences in sleep quality measures, specifically WASO and SE, were not evident in this sample and were not moderated by *APOE ϵ4* status. Instead, age emerged as the primary factor associated with sleep continuity, with older age linked to increased WASO and reduced SE, independent of sex and *APOE* genotype (see **Supplementary Table 1**).

**Figure 2:**
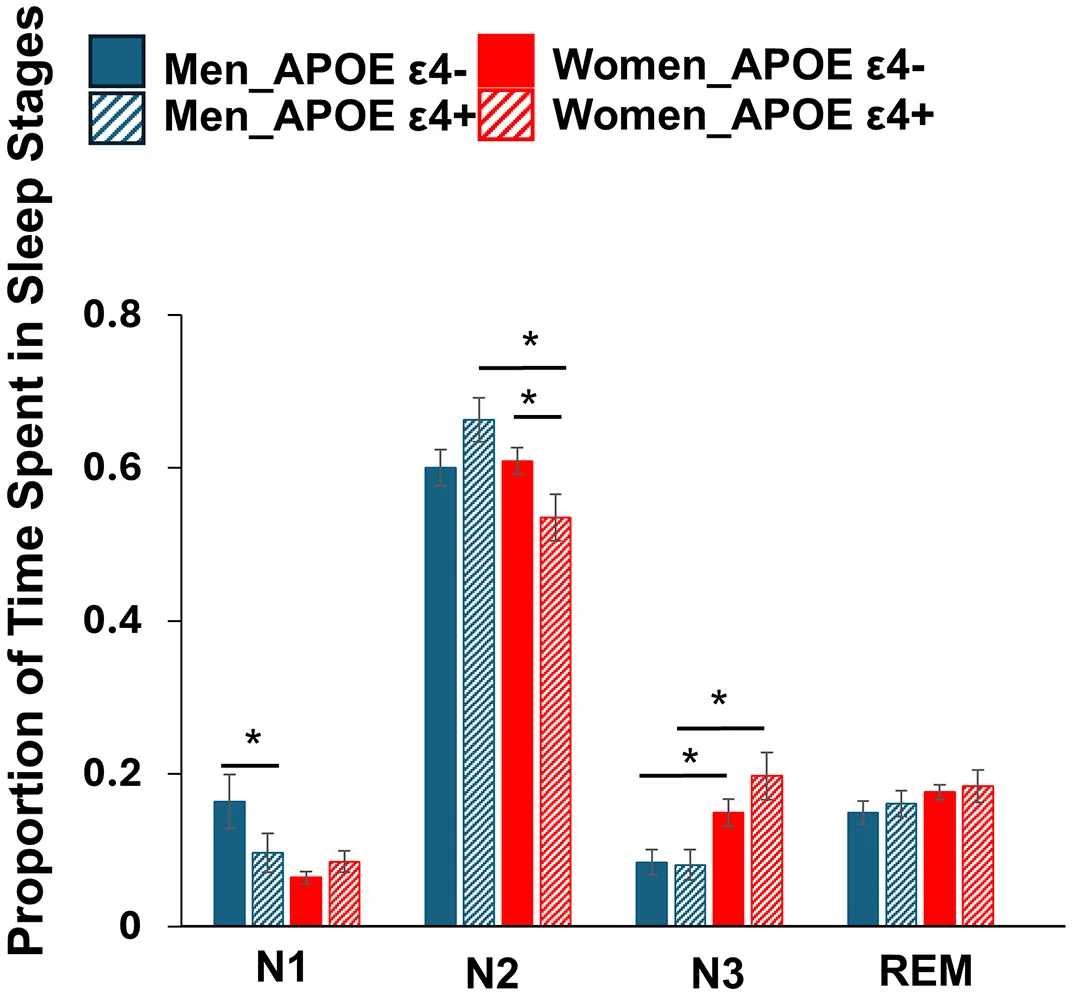
Sleep architecture measures stratified by sex and *APOE ϵ4* status. Percentage of total sleep time (TST spent in N1, N2, N3, and REM sleep is shown for men (blue) and women (red), with solid bars representing *APOE ϵ4* non-carriers and hatched bars representing *APOE ϵ4* carriers. Bars depict group means, and error bars represent ± SEM. Asterisks (*) indicate significant group differences (p <.05).

### 3.4 Sleep architecture and overnight memory retention

Given the observed sex differences in sleep architecture, we next examined whether associations between sleep architecture and overnight memory retention differed between men and women using Pearson correlations. In this sample, N2 sleep duration showed a nominal positive association with overnight memory retention in men (r = .40, p = .048), but this association did not survive FDR correction across sleep stages (q = .14). In contrast, this association was not significant in women (r = −.26, p = .11, q = .21), and the correlations differed significantly between sexes after FDR correction (z = 2.55, p = .005, q = .02), **Supplementary Figure 3A**. Further, N3 sleep duration was not significantly associated with overnight memory retention within either men or women, and neither association survived FDR correction. However, the N3-memory relationships differed significantly between sexes after FDR correction (z = 2.17, p = .010, q = .020), with women showing a more positive association than men, **Supplementary Figure 3B**. We did not observe a significant correlation between overnight memory retention and sleep duration for N1 or REM sleep (all p values>.07) nor were these associations significantly different from each other across men and women (all p values >.21).

When analyses were further stratified by *APOE ϵ4* status, among non-carriers we found no significant association between time spent in any sleep stage and overnight memory retention in men (all ps >.57) or women (all ps >.61). Among *APOE ϵ4* carriers, sleep-stage associations with overnight memory retention differed by sex. Among male APOE ϵ4 carriers, greater N2 sleep duration showed a nominal positive association with overnight memory retention (r = .68, p = .03), but this association did not survive FDR correction (q = .06), **Figure 3A**. The corresponding N2-memory association was not significant in female carriers (r = −.41, p = .23, q = .31). Despite the nonsignificant within-group associations after correction, the N2-memory correlations differed significantly between male and female *APOE* ϵ4 carriers after FDR correction (p = .009, q = .02).In contrast, greater N3 sleep duration showed a nominal positive association with overnight memory retention in female *APOE* ϵ4 carriers (r = .71, p = .02), but this within-group association did not survive FDR correction (q = .09), **Figure 3B**. The corresponding association was not significant in male carriers (r = −.51, p = .13, q = .17). However, the N3-memory correlations differed significantly between male and female *APOE* ϵ4 carriers after FDR correction (z = −2.71, p = .003, q = .01). Finally, greater REM sleep duration was associated with poorer overnight memory retention in male *APOE* ϵ4 carriers, and this association remained significant after FDR correction (r = −.78, p = .007, q = .03). The corresponding association in female carriers did not survive FDR correction (r = −.57, p = .075, q = .15), and the REM-memory correlations did not significantly differ between male and female carriers (z = .71, p = .24). Sensitivity analyses excluding the two ϵ4/ϵ4 homozygous participants preserved the significant between-sex differences in the N2- and N3-memory relationships among *APOE* ϵ4 carriers, as well as the negative REM–memory association in male carriers (**Supplementary Table S3**). No significant association between sleep quality (i.e., WASO and SE) and overnight memory retention were observed among participants (all ps >.29). Collectively, these findings suggest sex-specific patterns in associations between sleep architecture and overnight memory retention, particularly among *APOE ϵ4* carriers. Collectively, these findings suggest sex-specific patterns in associations between sleep architecture and overnight memory retention, particularly among *APOE* ϵ4 carriers. Although the individual N2-memory association in male carriers and N3-memory association in female carriers did not survive FDR correction, the corresponding correlation coefficients differed significantly between male and female *APOE* ϵ4 carriers after multiple comparisons correction. Specifically, the N2-memory relationship was more positive in male than female carriers, whereas the N3-memory relationship was more positive in female than male carriers. In addition, the negative association between REM sleep duration and overnight memory retention in male *APOE* ϵ4 carriers remained significant after FDR correction. These *APOE*-stratified findings remain exploratory given the modest subgroup sample sizes and warrant replication in larger samples.

**Figure 3:**
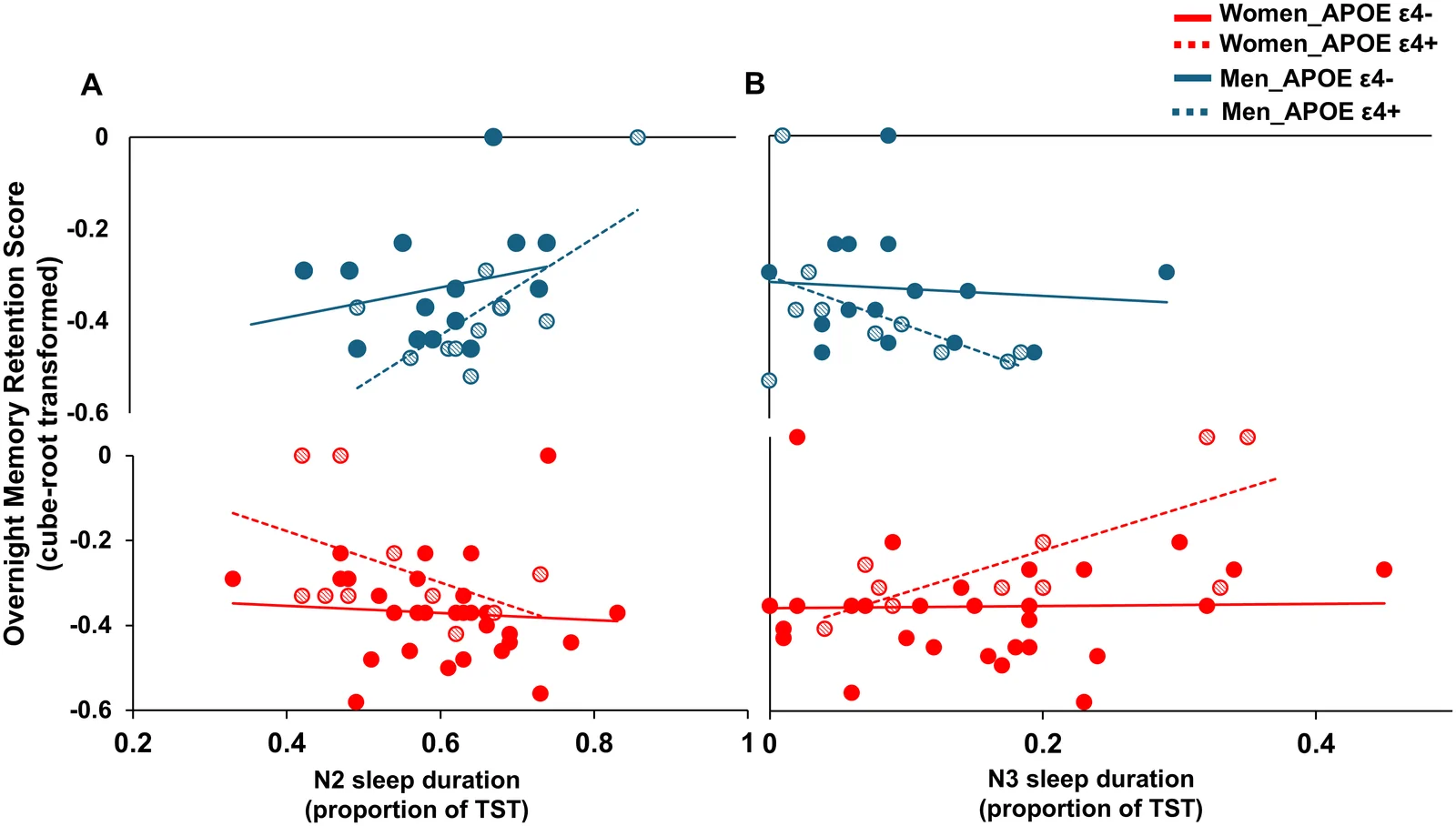
Associations between NREM sleep stages (N2, N3) and overnight memory retention stratified by sex and APOE ϵ4 status. **(A)** Relationship between N2 sleep duration (proportion of total sleep time, TST) and the cube-root-transformed proportional overnight memory retention score. **(B)** Relationship between N3 sleep duration (proportion of TST) and the cube-root-transformed proportional overnight memory retention score. Blue symbols denote men (top panel) and red symbols denote women (bottom panel). Solid lines represent *APOE* ϵ4 non-carriers and dashed lines represent *APOE* ϵ4 carriers. Points represent individual participants. Overnight memory retention was calculated as proportional change from immediate to delayed recall [(Delayed Recall − Immediate Recall)/Immediate Recall] and cube-root transformed to address non-normality. Plotted values, regression lines, and statistical analyses are based on the cube-root-transformed proportional overnight memory retention scores. Corresponding R^2^ values are: Men ϵ4− (N2 = .08; N3 = .01), Men ϵ4+ (N2 = .50*; N3 = .26), Women ϵ4− (N2 = .007; N3 = .0009), and Women ϵ4+ (N2 = .20; N3 = .53*).

### 3.5 Sex, *APOE ϵ4* status, and NREM sleep neurophysiology

We next examined whether local expression of NREM spectral activity differed by sex. We observed that, relative to men, women demonstrated greater global delta band power (1-4.5 Hz; p=.01; **Figure 4A**), with delta power at electrodes over central and parietal regions surviving TFCE correction. Women also exhibited significantly higher slow sigma power over central electrodes following TFCE correction for multiple comparisons (11–13 Hz; p=.02; **Figure 4B**). Next, analyses stratified sex effects by *APOE* ϵ4 status to explore whether *APOE* ϵ4 contributed to the observed sex differences in NREM spectral power. Among *APOE* ϵ4 carriers, women showed nominally greater central delta activity than men; however, this finding did not survive TFCE correction, **Figure 4**, and is therefore considered exploratory and not statistically significant after correction for multiple comparisons. Together, these exploratory analyses suggest that the overall sex differences in NREM spectral power were not consistently detected after stratification by *APOE* ϵ4 status. Given the smaller subgroup sample sizes, these stratified findings should be interpreted cautiously and may reflect reduced statistical power rather than true attenuation of sex-related differences.

**Figure 4:**
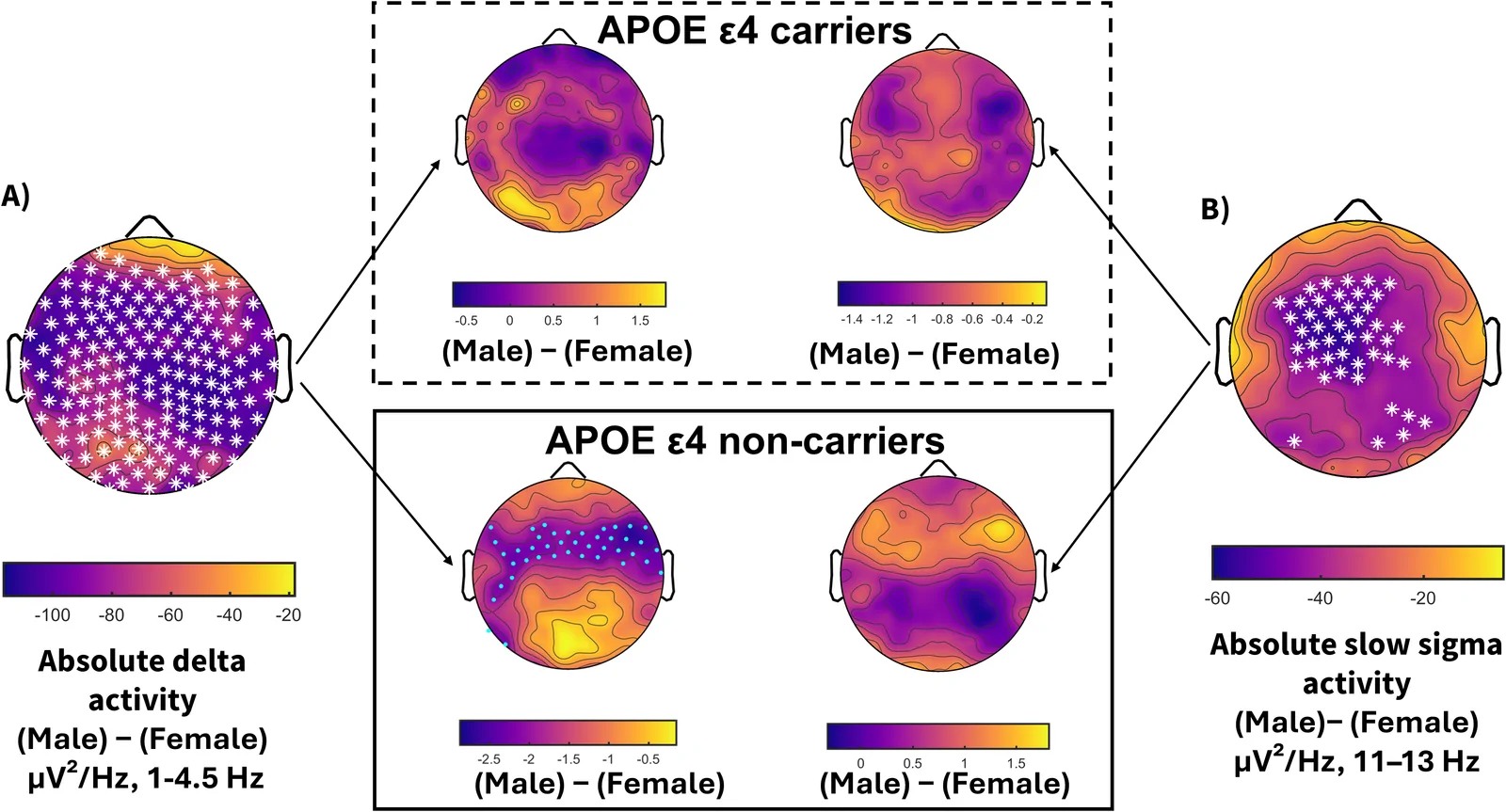
Sex-related differences in NREM spectral power by *APOE* ϵ4 status. Topographic maps depict sex differences in NREM spectral power computed as male minus female. **(A)** Sex differences in absolute delta activity (1-4.5 Hz) are shown for the full sample, with follow-up maps stratified by *APOE* ϵ4 carrier status. **(B)** Sex differences in absolute slow sigma activity (11–13 Hz) are shown for the full sample, with follow-up maps stratified by *APOE* ϵ4 carrier status. Warmer colors indicate higher spectral power in men relative to women, whereas cooler colors indicate higher spectral power in women relative to men. White asterisks denote electrodes surviving Threshold-Free Cluster Enhancement (TFCE) correction for multiple comparisons (p <.05). Cyan dots indicate electrodes reaching uncorrected p <.05 for visualization purposes only. Statistical inference is based on TFCE-corrected electrode-level results. Scalp orientation is shown with the nose at the top. Spectral power is expressed in μV^2^/Hz.

### 3.6 Sex, APOE, NREM sleep neurophysiology, and overnight memory associations

Lastly, we examined whether regional NREM spectral power was associated with overnight memory retention. In women, positive associations between overnight memory retention and both frontal slow sigma (p = .03) and fronto-central fast sigma power (p = .03) survived TFCE correction for multiple comparisons across the scalp at the electrodes indicated by white asterisks in **Figure 5A**. Cyan markers in **Figure 5A** indicate electrodes meeting the uncorrected threshold of p <.05 and are shown for exploratory visualization only. Spectral power was then averaged across the TFCE-significant electrodes and examined in partial correlations adjusting for age, years of education, and AHI. Both associations remained significant after covariate adjustment (slow sigma: partial r = .50, p = .002; fast sigma: partial r = .47, p = .004). No significant associations between overnight memory retention NREM spectral power in any band were observed in men (all p values>.10). Given that women showed higher sigma power and stronger sigma–memory associations, we next compared the frontal slow sigma–memory associations between women and men. Consistent with our *a priori* directional hypothesis, the association was stronger in women than in men in a one-tailed Fisher’s r-to-z comparison (p = .05).

**Figure 5:**
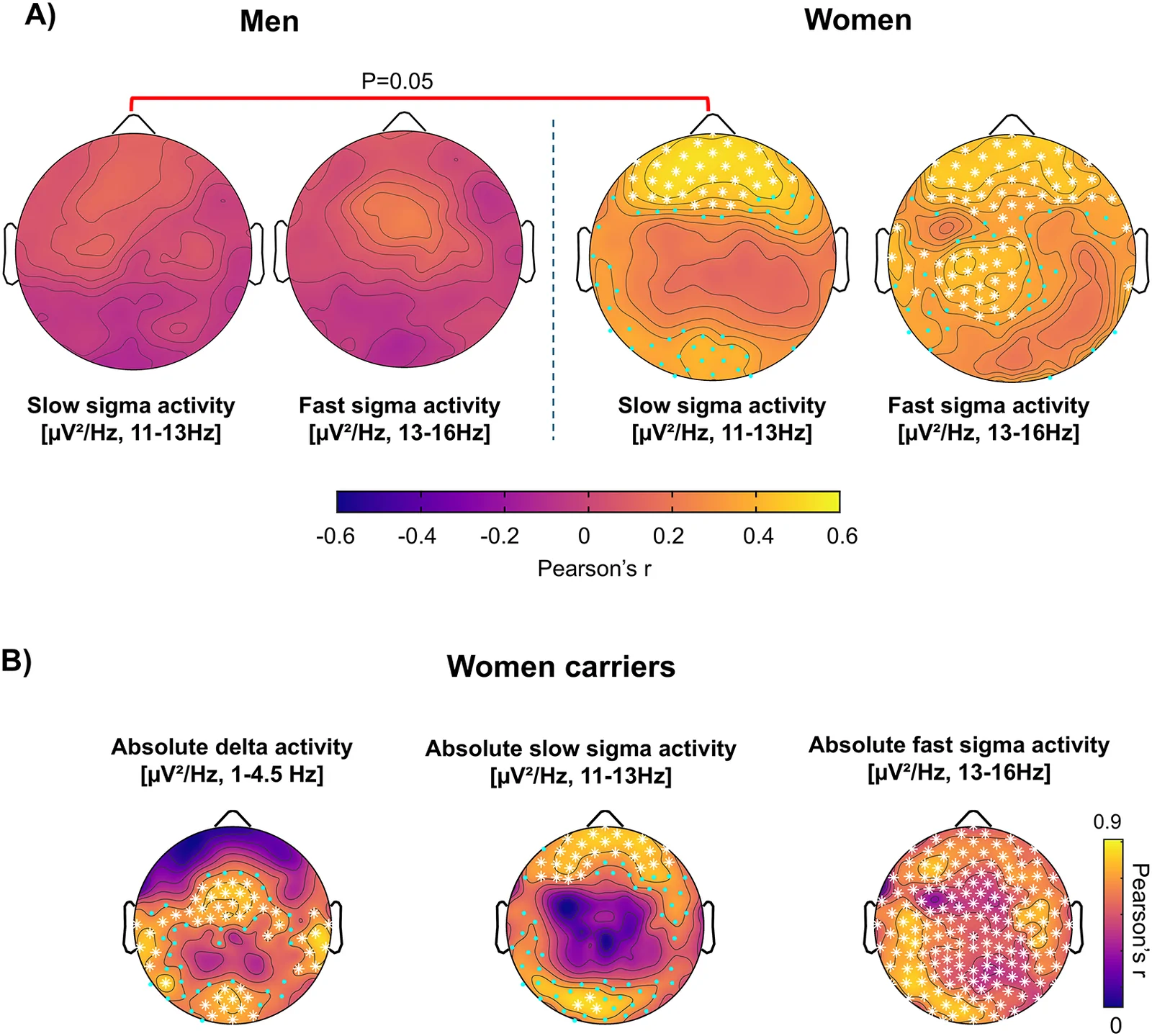
Topographic associations between NREM spectral power and overnight memory change by sex and *APOE ϵ4* status. **(A)** Associations between memory change and slow sigma activity (11–13 Hz) and fast sigma activity (13–16 Hz) are shown separately for men and women. Women showed stronger positive associations between sigma activity and memory, particularly over frontal regions. The bracket indicates a significant sex difference in the slow sigma–memory correlation strength based on Fisher’s r-to-z comparison (p = .05, one-tailed, based on an *a priori* directional hypothesis). **(B)** Among women *APOE* ϵ4 carriers, associations are shown for delta activity (1–4.5 Hz), slow sigma activity (11–13 Hz), and fast sigma activity (13–16 Hz). Warmer colors indicate stronger positive associations, whereas cooler colors indicate weaker or negative associations. Cyan dots indicate electrodes reaching the uncorrected threshold of p <.05 and are shown for exploratory visualization of the spatial distribution of associations only. White asterisks indicate electrodes surviving TFCE correction for multiple comparisons at p <.05.Statistical inference is based on TFCE-corrected electrode-level results unless otherwise specified. Scalp orientation is shown with the nose at the top. Spectral power is expressed in μV^2^/Hz.

We next examined whether these associations differed as a function of sex and *APOE ϵ4* status. Among female *APOE ϵ4* carriers, positive associations with slow sigma, fast sigma, and delta activity survived TFCE correction at the electrodes indicated by white asterisks in **Figure 5B**. Follow-up partial correlations of power averaged across these TFCE-significant electrodes, adjusting for age, education, and AHI, remained significant for slow sigma (partial r = .85, p = .01), fast sigma (partial r = .94, p = .001), and delta (partial r = .93, p = .003). Cyan markers indicate electrodes meeting the uncorrected threshold of p <.05 and are shown for exploratory visualization only. In contrast, no significant associations between NREM spectral activity and overnight memory retention were observed among *APOE* ϵ4 non-carriers or among men, irrespective of *APOE* ϵ4 status. Sensitivity analyses additionally adjusting for total sleep time preserved the overall pattern of NREM spectral power–memory associations (**Supplementary Table S4**).

## 4 Discussion

The central contribution of this study is the identification of sex-specific patterns in NREM sleep physiology and sleep-memory relationships in cognitively unimpaired older adults at increased risk for Alzheimer’s disease. Using PSG with hdEEG and an overnight word-pairs memory paradigm, we observed that sleep expression and the relationships between sleep expression and overnight memory retention differed by sex in older adults. Specifically, N3 duration was longer and NREM delta and slow sigma activity were higher in women relative to men, with sigma activity positively predicting overnight memory retention in women but not men. Further exploratory *APOE ϵ4-*stratified analyses showed that sleep-memory associations may be particularly relevant for women who are *APOE ϵ4* carriers. Exploratory *APOE* ϵ4-stratified analyses further indicated sex differences in the relationships of N2 and N3 sleep duration with overnight memory retention. Although the corresponding within-sex subgroup correlations did not individually survive FDR correction, the differences between male and female carrier correlations remained significant after correction. Importantly, these sleep-memory associations differed significantly by sex, indicating that distinct sleep neurophysiological features drive overnight memory retention in older men and women at risk for AD. Collectively, these findings suggest that sex-related variation in NREM sleep physiology may shape how sleep supports memory processes in aging and that *APOE ϵ4* status may further modify these relationships. In particular, oscillatory features linked to delta and sleep spindles may contribute to variability in sleep-dependent memory resilience particularly among older women at genetic risk for AD. However, given the modest subgroup sample size and exploratory nature of analyses stratifying across sex and *APOE ϵ4* status, these findings should be interpreted as preliminary and warrant replication in larger cohorts.

### 4.1 Sex-specific effects of *APOE ϵ4* on memory retention

While sex-related differences in overnight memory retention were not observed in the overall group, exploratory analyses suggested that among women, *APOE ϵ4* carriers exhibited less overnight forgetting relative to female non-carriers, whereas *APOE ϵ4* status was not associated with overnight memory retention in men. Moreover, male *ϵ4* carriers exhibited poorer memory retention than their female counterparts, suggesting that *APOE ϵ4*-related differences in overnight sleep-dependent memory retention may vary by sex, rather than reflecting a uniformly deleterious effect. Prior work indicates that *APOE ϵ4* effects can be age-dependent and pleiotropic, with younger or preclinical carriers sometimes showing preserved or even enhanced cognitive performance before later decline (2, 9, 58–62). This sex-specific pattern (60) is particularly interesting because *ϵ4* carriers are typically considered at greater risk for cognitive decline. The apparently favorable memory pattern observed here may reflect compensatory or resilience-related processes occurring during an early or preclinical stage of AD risk, potentially consistent with cognitive reserve frameworks (61, 62). However, this interpretation should be considered alongside alternative explanations. The pattern may be specific to cognitively unimpaired individuals before measurable cognitive decline has emerged, and it may also reflect selection or healthy volunteer effects, such that cognitively intact *APOE* ϵ4 carriers who participate in intensive research protocols represent a relatively high-functioning subset of individuals at genetic risk. Because the present study is cross-sectional, we cannot determine whether this pattern reflects transient early-stage preservation, a resilience-related process, stable interindividual variability, or a trajectory that precedes later cognitive decline. One possibility is that compensatory or resilience-related processes may transiently support preserved memory performance prior to later decline (2, 10, 63). However, sensitivity analyses indicated that the interaction persisted after adjustment for immediate-recall performance and that immediate recall itself did not differ significantly across sex or APOE ε4 groups. Replication using complementary approaches to characterizing memory change remains warranted. These findings are consistent with a recent report showing that slow wave activity may be more predictive of memory performance in older adults with greater amyloid-β burden (64), raising the possibility that memory-relevant sleep oscillations may contribute to preserved cognitive function in some individuals at elevated AD risk. Importantly, our memory findings co-occurred with sex-specific patterns in NREM sleep physiology, including greater N3 sleep and stronger sigma-memory associations, particularly among female *APOE ϵ4* carriers while N2 duration was longer and more memory relevant for male *APOE ϵ4* carriers. Together, these findings raise the possibility that enhanced NREM oscillatory activity may contribute to compensatory or resilience-related processes supporting preserved memory function in some cognitively unimpaired women at genetic risk for AD, although this interpretation cannot be established from the present cross-sectional data. These interpretations remain speculative and require longitudinal validation to determine their relevance for cognitive decline.

### 4.2 Sex differences in sleep architecture and their relation to *APOE ϵ4* status

Our findings suggest sex-specific patterns in sleep architecture and sleep-memory relationships that vary by *APOE ϵ4* status. Women spent a greater proportion of the night in N3 sleep overall, whereas men exhibited greater N2 sleep duration. Exploratory analyses further suggested that sex-related differences in N2 sleep varied modestly across *APOE ϵ4* status group. In contrast, *APOE ϵ4* status was not associated with N3 duration and did not interact with sex. These findings are consistent with prior work demonstrating robust sex differences in NREM sleep architecture across aging, with women generally exhibiting greater preservation of SWS relative to men (21, 22). Although hormone levels were not assessed in the present study, lifetime hormonal exposure and menopausal transition-related changes in sleep physiology may contribute to persistent sex differences in NREM sleep across aging (65–67).

Of note, these findings do not suggest that greater N3 sleep universally supports memory consolidation in older adults. Rather, consistent with prior work demonstrating an age-related decoupling between slow wave sleep and episodic memory, associations between N3 duration and memory retention in the present study were limited and context-dependent (14, 24, 68). This pattern aligns with evidence suggesting that increases in slow wave activity in later life may reflect altered or potentially excessive synaptic downscaling rather than enhanced consolidation, particularly in the context of AD risk (24, 25, 69).

These findings also extend the prior population-based work linking *APOE ϵ4* status to alterations in slow wave sleep, including reports of increased N3 duration among ϵ4/ϵ4 homozygous men in the Osteoporotic Fractures in Men (MrOS) cohort (29). In contrast to those studies, the present sample was composed predominantly of *ϵ4* heterozygotes, with few *ϵ4* homozygotes, limiting our ability to examine genotype dose effects. Consistent with Tranah et al., we did not observe differences in N3 sleep duration between *ϵ4* heterozygote carriers and non-carriers among men (29). Importantly, by including women and examining sleep-memory associations in addition to sleep stage duration, our results suggest that in predominantly heterozygous samples, sex-specific effects of *APOE ϵ4* may be more apparent in the functional relevance of sleep physiology for memory than in sleep architecture alone.

### 4.3 Sex and *APOE ϵ4* interact to influence sleep EEG-memory associations

Consistent with observed sex differences in sleep architecture (70–72), associations between NREM spectral activity and memory were also sex-dependent. In this sample, positive associations between spindle-relevant slow and fast sigma power and overnight memory retention were observed primarily among female *APOE ϵ4* carriers, whereas no such associations were evident in males or *ϵ4* non-carriers. These findings extend prior work from this cohort demonstrating that NREM sigma activity is associated with AD-related biomarkers, including inflammatory and tau-related processes (73). Notably, many of these biomarkers tend to be elevated in women and among individuals at increased AD risk, raising the possibility that sigma-related sleep physiology may play a distinct role in supporting memory function in women at genetic risk for AD. Although these associations were robust at the descriptive level, their attenuation in covariate-adjusted models warrants caution in inferring specificity beyond shared variance with demographic and sleep-related factors and suggests they should be interpreted as hypothesis-generating. These patterns may reflect sex-dependent effects of *APOE ϵ4* status (74) on sleep-related memory physiology in the face of elevated AD risk (2, 14, 61). These findings align with prior work linking sleep spindles to memory consolidation and suggest that sex-specific neural mechanisms may modulate their functional relevance (14, 19, 21, 75). Sigma band activity, which reflects sleep spindle dynamics, has been repeatedly implicated in hippocampal-cortical communication and synaptic plasticity (19, 20, 75, 76). The specificity of sigma-memory associations to female *APOE ϵ4* carriers suggests that spindle related mechanisms may be particularly sensitive to the interaction between sex biology and genetic risk (21, 24, 25).

Sleep stages are defined by their underlying oscillatory dynamics. These findings suggest that variability in slow waves and spindles, rather than stage labels alone, may provide greater mechanistic insight into sleep-memory relationships than sleep stage duration alone. Although effects may be detectable with conventional EEG, hdEEG provides greater spatial sensitivity to subtle regional variation in sleep neurophysiology that may relate to sex biology and genetic risk.

### 4.4 Implications and future directions

Together, these results highlight the importance of considering sex as a primary biological variable when studying mechanistic pathways linking sleep, cognition, and AD risk. Several studies have suggested that *APOE* effect may vary as a function of age and sex, and understanding these interactions may help clarify why women exhibit greater vulnerability to AD-related dementia (77–79). The present findings also raise the possibility that interventions targeting NREM oscillatory activity may have differential efficacy depending on sex and *APOE ϵ4* status, highlighting the potential value of precision approaches to AD prevention. Although the *APOE*-related findings should be interpreted cautiously given subgroup sizes, the results suggest that heterozygous *ϵ4* carriage may confer subtle and potentially sex-dependent neurophysiological effects that are not fully captured when sex is treated only as a covariate or when analyses are conducted in predominantly male samples (2, 9, 14, 80, 81). Future studies with larger samples will be important for determining whether sex-specific sleep-neurophysiology pathways contribute to differential AD vulnerability among *APOE ϵ4* carriers.

### 4.5 Limitations

Several limitations warrant consideration. First, the modest sample size, particularly within sex-by-genotype subgroups, limited power to detect subgroup-specific effects and interactions and may constrain generalizability. Additionally, a subset of participants was excluded from memory analyses due to missing data or predefined outlier criteria; however, included and excluded participants did not differ on key demographic or genetic variables, reducing concern for selection bias. Second, the cross-sectional design precludes causal inference or conclusions about temporal trajectories; longitudinal studies are needed to determine whether these sex-specific sleep-memory associations predict cognitive decline or AD-related pathology. Third, although analyses focused on NREM physiology, future work should more comprehensively examine REM sleep, given emerging evidence linking REM disruption to AD risk (82). Although we characterized NREM spectral activity, we did not quantify discrete sleep spindles, K-complexes, or slow waves; future event-based analyses are warranted. Accordingly, *APOE ϵ4*-related subgroup findings should be interpreted cautiously In addition, menopausal status and hormone therapy use were not assessed in this cohort. Given the potential influence of ovarian hormone status on sleep physiology and memory, we were therefore unable to evaluate whether these factors contributed to the observed sex differences. Future studies should directly characterize menopausal stage and exogenous hormone use. Because the study did not include a wake-control condition with a comparable retention interval, the observed memory changes cannot be attributed specifically to sleep. In addition, preclinical AD stage was not established for participants based on amyloid-β and tau biomarkers, thereby limiting interpretation of the observed patterns as evidence of compensation or resilience. Additionally, the study included multiple analyses across sleep measures and exploratory sex-by-*APOE* ϵ4 subgroups. Although TFCE correction was applied to electrode-level analyses and FDR correction was applied to related families of sleep stage-memory tests, the number of subgroup analyses and modest subgroup sizes increase the possibility of unstable effect estimates. Accordingly, *APOE* ϵ4-stratified findings should be considered hypothesis-generating and require replication in larger samples. Finally, the *APOE* ϵ4 carrier group combined heterozygous and homozygous carriers and the sample was not designed to assess *APOE* ϵ4 gene-dose effects.

### 4.6 Conclusion

In summary, sex differences in sleep physiology were evident in this sample and were associated with distinct patterns of sleep-memory relationships. Exploratory analyses further suggested that *APOE ϵ4* status may modify these relationships. These findings highlight the importance of incorporating sex as a primary biological variable in models of sleep, aging, and Alzheimer’s disease risk. Further work is needed to clarify the neurobiological basis and translational relevance of these interactions. Given the modest sample size, findings should be interpreted cautiously. Overall, the present results are consistent with the possibility that sex and genetic risk shape the relationship between NREM sleep physiology and memory function in older individuals at risk for dementia.

## Data Availability

The original contributions presented in the study are included in the article/**Supplementary Material**. Further inquiries can be directed to the corresponding authors.
