# Supplementary material for: Sex differences in sleep and memory relationships in older adults at risk for Alzheimer’s disease
Source: Front Endocrinol (Lausanne). 2026 Oct 12;17:1930177. doi: 10.3389/fendo.2026.1930177 (PMC13696260; doi:10.3389/fendo.2026.1930177)
Supplement: Supplementary file 1 [file SupplementaryFile1.docx]

Supplementary Materials

**Sex Differences in Sleep and Memory Relationships in Older Adults at Risk for Alzheimer’s Disease**

Negin Sattari, Abhishek Dave, Ivy Y. Chen, Kitty K. Lui, Miranda G. Chappel-Farley, Destiny E. Berisha, Kate E. Sprecher, Brady A. Riedner, Stephanie Jones, Barbara B. Bendlin, Bryce A. Mander, Ruth M. Benca

**Supplementary Results:**

**Sensitivity analysis adjusting for total sleep time.** To evaluate whether sleep quantity accounted for the observed NREM spectral power–memory associations, the partial correlations were repeated with TST included as an additional covariate, along with age, years of education, and AHI. Spectral power was averaged across the same TFCE-significant electrode clusters used in the primary analyses. In women, the association between overnight memory retention and frontal slow sigma remained significant after adjustment for TST (partial r = .45, p = .006), as did the association with fronto-central fast sigma power (partial r = .42, p = .01). Among female APOE ε4 carriers, the associations between overnight memory retention and slow sigma (partial r = .79, p = .05), fast sigma (partial r = .97, p = .001), and delta power (partial r = .88, p = .02) also remained significant after inclusion of TST. Detailed results of these sensitivity analyses are summarized in Supplementary Table S4.

**Sensitivity analysis excluding APOE ε4/ε4 homozygotes.** To determine whether the principal APOE-related findings were disproportionately influenced by the two ε4/ε4 homozygous participants, the Figure 1 and Figure 3 analyses were repeated after excluding these individuals. The principal findings were preserved; results are summarized in Supplementary Table S3.

**Sensitivity analyses addressing immediate-recall performance.** Because proportional change scores may be influenced by baseline performance, we conducted additional analyses to evaluate whether immediate-recall performance contributed to the observed sex × APOE ε4 interaction in proportional overnight retention. First, immediate recall was not significantly correlated with proportional overnight retention (r = .11, p = .39), indicating that initial recall performance was not systematically associated with the magnitude of proportional overnight change.

Second, the primary ANCOVA was repeated with proportional overnight retention as the dependent variable and immediate recall included as an additional covariate, together with sex, APOE ε4 status, the sex × APOE ε4 interaction, age, AHI, and years of education. The sex × APOE ε4 interaction remained significant, F(1,56) = 4.05, p = .049, partial η² = .067, whereas immediate recall was not significantly associated with proportional overnight retention, F(1,56) = 0.10, p = .755, partial η² = .002.

Third, we examined whether immediate-recall performance itself differed by sex or APOE ε4 status. In an ANCOVA with immediate recall as the dependent variable and age, AHI, and years of education as covariates, there was no significant main effect of sex, F(1,60) = 0.66, p = .421, partial η² = .011; no significant main effect of APOE ε4 status, F(1,60) = 0.01, p = .943, partial η² < .001; and no significant sex × APOE ε4 interaction, F(1,60) = 1.17, p = .284, partial η² = .019. Together, these analyses do not support the interpretation that the observed sex × APOE ε4 interaction in proportional overnight retention was driven by systematic differences in immediate-recall performance.

Supplementary Figure 1: Overnight memory retention as a function of sex. Bars represent mean cube-root-transformed proportional overnight memory retention scores for men (blue) and women (pink). More negative values indicate greater overnight forgetting. Error bars represent the standard error of the mean (SEM). No significant sex differences in overnight memory retention were observed.

Supplementary Figure 2: Sex differences in sleep architecture. Bars represent the mean proportion of total sleep time spent in each sleep stage (N1, N2, N3, and REM) for men (blue) and women (pink). Women exhibited a significantly greater proportion of N3 sleep relative to men (p < .05). Error bars represent standard error of the mean (SEM).

Supplementary Figure 3: Associations between sleep architecture and overnight memory retention by sex. Scatterplots depict associations between the proportion of total sleep time (TST) spent in N2 (A) and N3 (B) sleep and the cube-root-transformed proportional overnight memory retention score, separately for men (blue) and women (pink). Overnight memory retention was calculated as proportional change from immediate to delayed recall [(Delayed Recall − Immediate Recall) / Immediate Recall] and cube-root transformed to address non-normality. (A) Greater N2 sleep duration was positively associated with overnight memory retention in men, whereas no significant association was observed in women; the N2–memory correlation coefficients differed significantly between men and women. (B) Although the associations between N3 sleep duration and overnight memory retention were not individually significant within either sex group, the N3–memory correlation coefficients differed significantly between men and women. Lines represent linear regression fits using the cube-root-transformed proportional overnight memory retention scores. Asterisks indicate significant between-sex differences in correlation coefficients at p < .05.

Supplementary Table S1. Results for Sleep Quality Measures

| Model | Outcome | Overall p-value | Significant covariate effect | Sex | APOE ᶓ4 | | Sex × APOE ᶓ4 | Interpretation |
| --- | --- | --- | --- | --- | --- | --- | --- | --- |
| Sex model | WASO | .01 | Age:  F(1,75) = 8.65, p = .004,  partial η² = .103 | p = .38 | — | — | | Older age was associated with increased WASO; no sex effect was observed. |
| Sex model | Sleep efficiency | .06 | Age:  F(1,75) = 6.98, p = .010,  partial η² = .085 | p = .75 | — | — | | Older age was associated with lower sleep efficiency; no sex effect was observed. |
| Sex + APOE ε4 model | WASO | .04 | Age:  F(1,73) = 9.04, p = .004,  partial η² = .110 | p = .47 | p = .43 | F(1,73) = 0.01,  p = .90,  partial η² < .001 | | Older age was associated with increased WASO; no sex, APOE ε4, or interaction effects were observed. |
| Sex + APOE ε4 model | Sleep efficiency | .18 | Age:  F(1,73) = 6.96, p = .010,  partial η² = .080 | p = .91 | p = .75 | F(1,73) = 0.16,  p = .69,  partial η² = .002 | | Older age was associated with lower sleep efficiency; no sex, APOE ε4, or interaction effects were observed. |

Results from univariate ANCOVA models examining sleep quality measures are summarized in Table 1. Across models, age was the only significant predictor of WASO and sleep efficiency, with older age associated with increased WASO and lower sleep efficiency. No significant effects of sex, *APOE* ε4 status, or sex × *APOE* ε4 interactions were observed.

**Supplementary Table S2. Neuropsychological performance of the study sample**

**N = 80**

| **Measure** | **Mean ± SD** |
| --- | --- |
| Interval from assessment to PSG (years) | 0.7 ± 0.7 |
| Montreal Cognitive Assessment | 27.6 ± 2.1 |
| **Craft Story** |  |
| Immediate recall (verbatim, total units) | 24.4 ± 5.6 |
| Delayed recall (verbatim, total units) | 21.5 ± 6.0 |
| Immediate recall (paraphrase, total units) | 17.4 ± 3.4 |
| Delayed recall (paraphrase, total units) | 16.6 ± 3.9 |
| **Benson Figure** |  |
| Immediate recall | 15.8 ± 0.9 |
| Delayed recall | 13.2 ± 2.3 |
| Multilingual Naming Test | 30.6 ± 1.8 |
| **Phonemic Fluency Test** |  |
| Letter fluency (total F words in 60 s) | 16.4 ± 4.4 |
| Letter fluency (total L words in 60 s) | 15.4 ± 4.2 |
| Letter fluency (total F and L words in 60 s) | 31.8 ± 7.4 |
| Category fluency (vegetable words in 60 s) | 16.8 ± 4.2 |
| **Trail Making Test** |  |
| Trails A (time in s) | 21.8 ± 8.1 |
| Trails B (time in s) | 55.3 ± 30.0 |

Note. Values are mean ± sample SD. Interval is the absolute difference between neuropsychological assessment and PSG ages. Animal fluency and Number Span measures were not available in the supplied dataset. PSG = polysomnography.

**Supplementary Table S3. Sensitivity analyses excluding APOE ε4/ε4 homozygotes**

**Panel A. Sex × APOE ε4 interaction on proportional overnight memory retention**

| **Analysis** | **Effect** | **F** | **df** | **p** | **Partial η²** |
| --- | --- | --- | --- | --- | --- |
| Primary analysis | Sex × APOE ε4 | 7.36 | 1, 58 | .009 | .116 |
| Excluding ε4/ε4 homozygotes | Sex × APOE ε4 | 4.64 | 1, 56 | .036 | .078 |

**Panel B. Principal APOE ε4-carrier sleep-stage–memory findings after exclusion of ε4/ε4 homozygotes**

| **Finding** | **Group / comparison** | **Statistic** | **p** | **q** | **Primary analysis** | **Sensitivity interpretation** |
| --- | --- | --- | --- | --- | --- | --- |
| N2–memory sex difference | Male vs female APOE ε4 carriers | z = 3.15 | .002 | .003 | Significant after FDR correction | Preserved |
| N3–memory sex difference | Male vs female APOE ε4 carriers | z = −3.27 | .001 | .003 | Significant after FDR correction | Preserved |
| REM–memory association | Male APOE ε4 carriers | r = −.761 | .028 | .037 | Significant after FDR correction | Preserved |

Note. APOE = apolipoprotein E; FDR = false discovery rate. Panel A models included age, apnea-hypopnea index, and years of education as covariates. The sensitivity analyses excluded the two APOE ε4/ε4 homozygous participants. Panel B summarizes the principal Figure 3 findings that were significant in the primary analysis and remained significant after exclusion.

**Supplementary Table S4. Sensitivity analyses of NREM spectral power–memory associations adjusting for total sleep time**

| **Group** | **Frequency band / region** | **Original partial r** | **Original p** | **TST-adjusted partial r** | **TST-adjusted p** | **Interpretation** |
| --- | --- | --- | --- | --- | --- | --- |
| Women | Frontal slow sigma | .50 | .002 | .45 | .006 | Preserved |
| Women | Fronto-central fast sigma | .47 | .004 | .42 | .010 | Preserved |
| Female APOE ε4 carriers | Slow sigma | .85 | .010 | .79 | .050 | Preserved / threshold-level |
| Female APOE ε4 carriers | Fast sigma | .94 | .001 | .97 | .001 | Preserved |
| Female APOE ε4 carriers | Delta | .93 | .003 | .88 | .020 | Preserved |

**Note.** Original partial correlations adjusted for age, years of education, and apnea-hypopnea index (AHI). Sensitivity analyses additionally adjusted for total sleep time (TST). Spectral power was averaged across the same TFCE-significant electrode clusters identified in the primary analyses. The slow sigma association in female APOE ε4 carriers was at the conventional significance threshold after TST adjustment (p = .050).
